# Chemical and Enantioselective Analysis of the Leaf Essential Oil from *Piper coruscans* Kunth (Piperaceae), a Costal and Amazonian Native Species of Ecuador

**DOI:** 10.3390/plants9060791

**Published:** 2020-06-24

**Authors:** Gianluca Gilardoni, Yadira Matute, Jorge Ramírez

**Affiliations:** Departamento de Química y Ciencias Exactas, Universidad Técnica Particular de Loja, Calle M. Champagnat s/n, Loja 1101608, Ecuador; gianluca.gilardoni@gmail.com (G.G.); yadiram6644@gmail.com (Y.M.)

**Keywords:** *Piper coruscans*, *Artanthe amazonica*, *Piper amazonicum*, essential oil, GC-MS, GC-FID, enantioselective analysis, Ecuador

## Abstract

In the present study, an essential oil was distilled from the leaves of *Piper coruscans* Kunth, a native Amazonian species belonging to the family Piperaceae and quite common in Ecuador. The chemical analysis was performed by GC-MS (qualitative) and GC-FID (quantitative), on polar and non-polar columns, detecting a total of 58 compounds of which 52 were identified. All the identified compounds were quantified. The essential oil was mainly constituted of sesquiterpenes (54.1–55.0%) and oxygenated sesquiterpenoids (32.5–33.6%), the major constituents being: (*E*)-β-caryophyllene (24.1–25.0%), α-humulene (11.6–12.0%), caryophyllene oxide (9.3–10.9%), linalool (4.5–5.2%), humulene epoxide II (3.6–4.1%), (*E*)-nerolidol (3.7–4.0%), α-copaene (3.7–3.9%), α-muurolol (3.4–3.7%), α-selinene (3.4–3.5%), β-selinene (3.1–3.3%), and one undetermined oxygenated sesquiterpenoid (3.1–3.3%). The aqueous phase (hydrolate) of the distillation process was also submitted to chemical analysis, showing linalool as the main organic compound in solution, with a concentration of 12.3–15.7 mg/100 mL. The essential oil was than analyzed for the enantiomeric distribution of its monoterpene constituents, affording the following enantiomeric excesses in two β-cyclodextrin-based enantioselective columns: (1*S*,5*S*)-(-)-α-pinene (60.0–69.6%), (1*S*,5*S*)-(-)-β-pinene (5.2–7.2%), (*R*)-(-)-α-phellandrene (72.5–78.2%), (*R*)-(+)-limonene (28.6%) and (*R*)-(-)-linalool (1.8–3.1%).

## 1. Introduction

Ecuador is a small country located across the Equatorial line, overlooking the Pacific Ocean in the northern portion of the South American continent. It is geographically and climatically divided in four main regions: The islands (Galapagos), the coast, the Andean region, and the Amazonian forest. Each one of these zones is characterized by a peculiar climate, what makes biodiversity an incredible strength for the country. That is why Ecuador has been recognized by the UN Environment Program World Conservation Monitoring Centre as one of the 17 megadiverse countries in the world, counting by definition with “at least 5000 of the world’s plants as endemics” [1].

Every year, botanists discover and describe in Ecuador new botanical species, that are added to the approximately 16,000 already known. According to the most complete botanical publication on the Ecuadorian flora [2], 15,306 native species were known in 1999, of which 4173 were endemic. Most of the native plants described in this country have never been investigated so far for what concerns their metabolic composition [3]. This is the reason why the authors have been studying for many years the secondary metabolites of the Ecuadorian flora, in order to give a contribution to the knowledge in phytochemistry and phytopharmacolgy.

Among the natural products the authors are interested in, we can cite essential oils (EOs) [4,5,6,7,8,9,10,11]. According to the European Pharmacopeia, an EO is an “odorous product, usually of complex composition, obtained from a botanically defined plant raw material by steam distillation, dry distillation, or a suitable mechanical process without heating. Essential oils are usually separated from the aqueous phase by a physical process that does not significantly affect their composition” [12]. In this context, the authors decided to describe the chemical and enantiomeric composition of the EO distilled from the leaves of *Piper coruscans* Kunth.

*Piper coruscans* is a species belonging to the family Piperaceae, described as a native plant of the coast and Amazonian regions of Ecuador and growing wild between 0–500 m above sea level [2]. Nevertheless, it has been described in many other countries, from French Guyana to Brazil, from Venezuela to Colombia, from Peru to Bolivia [13]. *Piper coruscans* is also known with many synonyms: *Artanthe amazonica* Miq., *Artanthe coruscans* (Kunth) Miq., *Artanthe pseudochurumayu* (Kunth) Miq., *Piper amazonicum* (Miq.) C. DC., *Piper baryanum* C. DC., *Piper coactaepilum* Trel., *Piper coruscans* var. *membranaceum* (C. DC.) Steyerm., *Piper orenocanum* C. DC., *Piper pseudochurumayu* (Kunth) C. DC., *Piper pseudochurumayu* var. *membranaceum* C. DC., *Piper santiaganum* Trel., *Piper tingens* Trel., *Piper wurdackii* Yunck., *Schilleria coruscans* (Kunth) Kunth, and *Steffensia pseudochurumayu* Kunth [13].

According to a recent comprehensive review on the phytochemistry of genus *Piper*, the leaves of *P. coruscans* are used in traditional medicine as a purgative. Furthermore, the decoction is considered effective to treat high fevers, whereas the warmed leaves are reduced to poultice to treat swollen abdomen in children [14].

Despite a lot of literature exists about the chemistry of genus *Piper*, only about 10% of all known *Piper* species have been submitted to a phytochemical study [14]. For what concerns *P. coruscans*, seven references have been found in literature. Six of them were related to the chemistry, synthesis, and biological activity of non-volatile extracts and metabolites (mainly coruscanones) [15,16,17,18,19], whereas only one cited the essential oil [20]. However, nowhere the chemical or the enantioselective analysis was reported. On the other hand, no literature was found about any synonyms of this species, whereby, to the best of the authors’ knowledge, this study describes for the first time an EO distilled from *Piper coruscans* Kunth.

## 2. Results

### 2.1. Chemical Analysis

The essential oil was obtained with a distillation yield of 0.4 ± 0.26% from fresh plant material. The chemical analyses were performed on two different columns, a non-polar one (DB-5ms) and a polar one (HP-INNOWax), detecting a total of 58 compounds. Most of the constituents (52) were identified by comparing the electron impact mass spectrum (EIMS) and the linear retention index (LRI) with literature, whereas 6 remained unidentified. According to their molecular weight, the unknown components are consistent with one sesquiterpene (204 amu) and five oxygenated sesquiterpenoids (220 and 222 amu). For what concerns the quantitative analysis, 46 identified constituents, corresponding to about 91% of the EO, could be quantified on at least one column, whereas 6 compounds (camphene, p-cymene, terpinen-4-ol, cyclosativene, β-cubebene, and aromadendrene) appeared as traces (<0.1%) in both columns. In this case, due to the abundance of oxygenated terpenoids, the existence of an important residual organic fraction dissolved in the aqueous phase (hydrolate) was supposed. Hence, the distillation water phase was analyzed in the same conditions of the EO, after concentration by solid phase extraction (SPE). The results were expressed as milligrams of analytes per 100 mL of water. The main organic substance in solution was linalool, with an abundance of 12.3–15.7 mg/100 mL. All the analytical results are reported in Table 1.

### 2.2. Enantioselective Analysis

The enantioselective analysis was performed on two enantioselective columns: a 2,3-diethyl-6-*tert*-butyldimethylsilyl-β-cyclodextrin and a 2,3-diacetyl-6-*tert*-butyldimethylsilyl-β-cyclodextrin based capillary columns. A total of five enantiomeric pairs were identified, all belonging to the class of monoterpenes and monoterpenoids. None of the detected chiral compounds was enantiomerically pure. The complete enantioelective analysis is represented in Table 2.

## 3. Discussion

According to literature [14], some authors classify the EOs distilled from the species of genus *Piper* into six categories, depending on their chemical composition: EOs dominated by monoterpenes (mainly limonene, sabinene, β-pinene, α-pinene, and piperitone), EOs dominated by sesquiterpenes (typically β-caryophyllene, germacrene D, β-elemene, *epi*-cubebol, β-guaiene, and β-bisabolene), EOs equally dominated by both families of terpenoids, EOs dominated by phenylpropanoids (for example safrole, dillapole, eugenol, chavibetol, and (*Z*)-asarone), EOs dominated by benzenoid compounds and EOs dominated by non-terpenoid compounds (usually derivatives from the acetate pathway). Observing the chemical analysis performed in the present study, we can conclude that the EO distilled from the leaves of *P. coruscans* clearly belongs to the second group. In fact, despite α-pinene, β-pinene and linalool were present in a significant amount, about 80% of the chemical composition corresponded to sesquiterpenes and sesquiterpenoids. In particular, (*E*)-β-caryophyllene (24.1–25.0%), α-humulene (11.6–12.0%), and caryophyllene oxide (9.3–10.9%) were clearly dominant. In this case, the plant material was freshly distilled after collection and the EO immediately injected, what makes the authors think that no artefact was significantly produced. However, caryophyllene oxide is sometime considered as a result of aging in a (*E*)-β-caryophyllene containing EO. If this were the case, the real amount of (*E*)-β-caryophyllene would overpass 30%. The very high content of (*E*)-β-caryophyllene makes *P. coruscans* EO relatively quite similar to the one obtained from fruits of *P. nigrum* (black pepper), where the abundance of this sesquiterpene ranges normally between 15–50% but it can rise until 70% in some Malaysian cultivars [14]. The high amount of (*E*)-β-caryophyllene also opens the way to the study of interesting biological properties for this EO, according to great number of bioactivities described in literature for this sesquiterpene [34].

For what concerns the aqueous phase that spontaneously separates from an EO after distillation, commonly called hydrolate, it is well known that sometimes it has an important commercial value, such is the case for example of rose water or mint water. For the EO of *P. coruscans*, the high content of oxygenated terpenoids suggested that an important residue could remain dissolved in water, which effectively presented a clear sweet odor. The chemical analysis of the hydrolate revealed that linalool is actually the very most abundant organic solute, reaching the concentration of about 15 mg/100 mL (150 ppm), what explains the perceived aroma.

The chemical analysis of this essential oil was complemented with the enantioselective one, where the enantiomeric distribution and the enantiomeric excess (*e.e.*) of some monoterpenes and monoterpenoids were determined and confirmed on two different enantioselective columns. None of the detected chiral metabolites was present in its enantiomerically pure form, however β-pinene and linalool were almost racemic, with just a small *e.e.* in favor of (1*S*,5*S*)-(-)-β-pinene and (*R*)-(-)-linalool.

## 4. Materials and Methods

### 4.1. Plant Material

The leaves of *P. coruscans* were collected on April 2018 in the province of Zamora-Chinchipe, near town Zamora, at coordinates 04°05′00′’ S and 078°57′00′’ W. The plant was collected under permission N° 001-IC-FLO-DBAP-VS-DRLZCH-MA, emitted by the Ministry of Environment of Ecuador. The species was identified by botanist Dr. Vladimir Morocho of the Universidad Técnica Particular de Loja (UTPL) and a voucher specimen was deposited at the herbarium of UTPL with code PPN-pi-010.

### 4.2. Distillation of the Essential Oil and Sample Preparation

In order to obtain the pure essential oil, 3 kg of fresh plant material were preparatively hydrodistilled for 4 h, inside a stainless-steel Clevenger-type apparatus. After recovery of the organic layer, the EO was dried over anhydrous sodium sulphate. For all the GC injections, 10 mg of EO were weighted and diluted with 1 mL of cyclohexane, previously prepared with an internal standard (*n*-nonane) at the concentration of 0.7 mg/mL. Additionally, four portions of 10 mL of the water layer were collected and eluted on previously conditioned solid phase extraction (SPE) columns. After complete removal of water from the solid phase, the analytes were recovered by elution with 2 mL of acetone prepared, as previously described for cyclohexane, dissolving *n*-nonane as internal standard (0.7 mg/mL). The acetone solutions were directly injected into GC. The SPE columns were standard products, packed with 1 g of C-18 reversed phase and purchased from Sigma-Aldrich. 

Additionally, four analytical repetitions were performed hydrodistilling the essential oil inside a micro-scale Marcusson-type apparatus [35]. In this case, 10 g of fresh plant material were distilled for 90 min and the volatile fraction was collected in 400 μL of an extractive organic layer (cyclohexane containing 0.7 mg/mL of *n*-nonane as internal standard). The cyclohexane layers were recovered and directly injected into GC.

In this study, all the samples were transferred to amber vials and kept at −15 °C until use. After verifying the similarity of the GC profile between preparative and analytical repetitions, all the five samples were used to calculate the mean distillation yield and afforded the mean quantitative results, both provided with standard deviation.

All the solvents used in this study (analytical grade, purity >99%) were purchased from Sigma-Aldrich.

### 4.3. Qualitative Chemical Analysis

The qualitative chemical analyses were performed with a gas chromatography-mass spectrometry (GC-MS) system, constituted by an Agilent Technologies gas chromatograph 6890N coupled to a simple quadrupole Mass Spectrometry Detector (MSD) model 5973 (Santa Clara, CA, USA). The MSD was operated in SCAN mode, with an electronic ionization source of 70 eV. The ion detection was limited to the range of 35–350 m/z. The transfer line was set at the temperature of 280 °C, the MS ion source at 200 °C. The gas chromatograph was configurated with a DB-5ms non-polar (5%-phenyl-methylpolysiloxane, 30 m, 0.25 mm internal diameter, and 0.25 μm film thickness; J & W Scientific, Folsom, CA, USA) and a HP-INNOWax polar (polyethylene glycol, 30 m, 0.25 mm internal diameter and 0.25 μm film thickness; Agilent Technologies, Santa Clara, CA, USA) capillary columns.

The GC-MS analyses on DB-5ms were performed as follow: the carrier gas was helium, set at constant flow, with a rate of 1 mL/min. All the chromatographic runs were performed injecting 1 μL. The injector was set in split mode (40:1), with an injection temperature of 250 °C. The elution was conducted from 50 °C (1 min) to 250 °C (10 min) at a gradient rate of 3 °C/min.

The same conditions and thermal program were used for the analyses on HP-INNOWax, except for the final temperature, that just reached 230 °C due to the lower thermal stability of the stationary phase.

In order to identify the components of the EO, the linear retention index (LRI) of each constituent was calculated according to Van Den Dool and Kratz [36] and compared to literature, together with the corresponding mass spectrum (see Table 1). LRIs were calculated through the homologous series of linear alkanes, using a mixture from *n*-nonane to *n*-pentacosane (*n*-nonane purity was 99% from BDH, Dubai, UAE. C_10_–C_25_ purity was 99% from Sigma-Aldrich, St. Louis, MO, USA).

### 4.4. Quantitative Chemical Analysis

The quantitative analyses were run in the same GC instrument as the qualitative ones, configured with a Flame Ionization Detector (FID) and equipped with an Agilent Technologies 7683 series autoinjector (Little Falls, DE, USA).

The analytical conditions were the same described for the qualitative analyses, but with a different thermal program. In fact, with DB-5ms column, the initial temperature of 50 °C was kept for 1 min, followed by a thermal gradient of 3 °C/min until 180 °C, then a second thermal gradient of 15 °C/min until 250 °C. The final temperature was maintained for 15 min. For what concerns the analysis on HP-INNOWax, the same GC method as DB-5ms was applied, except for the final temperature that only reached 230 °C. The FID was alimented with a mixture of hydrogen and air, at the flow of 30 mL/min and 300 mL/min respectively. The detector was set at the temperature of 250 °C. In order to quantify the analytes, a relative response factor (RRF) was calculated for each component, according to the respective combustion enthalpy [37,38]. In this respect, A. Chaintreau and colleagues demonstrated that the RRF of an organic compound, analyzed by FID, only depends, with good approximation, on its molecular formula and number of aromatic rings. According to this principle, they described a mathematical formula [38], that permits to estimate the RRF toward a quantification standard (usually methyl octanoate). In our case, a modified method was actually applied, since isopropyl caproate was used instead of methyl octanoate and two calibration curves (one for each column) have been used instead of a single point internal standard. The isopropyl caproate was prepared by synthesis in one of the authors’ laboratory (G.G.) and its purity was calculated by GC as 97%. For calibration curves construction, six calibration standard dilutions were prepared, dissolving 0.6, 1.8, 4.3, 8.3, 16.8, and 34.3 mg of isopropyl caproate in 10 mL of cyclohexane respectively. As usual, an amount of 7.0 mg of *n*-nonane was used as internal standard inside each dilution. Both calibration curves generated a correlation coefficient of 0.995.

### 4.5. Enantioselective GC Analysis

The enantioselective analyses were carried out in the same previously described GC-MS system, measuring the enantiomeric relative percentage and the enantiomeric excesses (*e.e.*). The instrument was equipped with a 2,3-diethyl-6-*tert*-butyldimethylsilyl-*β*-cyclodextrin and a 2,3-diacetyl-6-*tert*-butyldimethylsilyl-β-cyclodextrin enantioselective columns, both 25 m × 0.25 mm × film thickness 0.25 µm from Mega, Legnano, Italy.

The following thermal program was applied: 50 °C maintained for 5 min, then a gradient temperature of 2 °C/min until 220 °C, that were kept for 5 min. The enantiomer order of elution was determined through the injection, in the same instrumental conditions, of mixtures of enantiomerically pure standards.

## 5. Conclusions

The leaves of *Piper coruscans* Kunth contain a volatile fraction of prevalently sesquiterpene composition. The hydrodistillation of the leaves produces an essential oil, whose known major compounds are (*E*)-β-caryophyllene (24.1–25.0%), α-humulene (11.6–12.0%), caryophyllene oxide (9.3–10.9%), linalool (4.5–5.2%), humulene epoxide II (3.6–4.1%), (*E*)-nerolidol (3.7–4.0%), α-copaene (3.7–3.9%), α-muurolol (3.4–3.7%), α-selinene (3.4–3.5%), and β-selinene (3.1–3.3%). The sesquiterpene fraction of this EO counts for more than 80% of the chemical composition. For what concerns the monoterpene fraction, at least five of its chiral components subsist as mixtures of enantiomeric pairs.

## Figures and Tables

**Table 1:** Chemical analysis of *Piper coruscans* essential oil.

N.	Constituents					Essential Oil				Hydrolate			
	Identification	DB-5ms		HP-INNOWax		DB-5ms		HP-INNOWax		DB-5ms		HP-INNOWax	
		LRI ^1^	LRI ^2^	LRI ^1^	LRI ^3^	% ^4^	σ ^5^	% ^4^	σ ^5^	mg/100 mL	σ ^5^	mg/100 mL	σ ^5^
1	α-pinene	925	932	1014	1028 ^6^	2.4	0.77	3.0	0.94	-	-	-	-
2	camphene	936	946	1056	1075 ^6^	trace	-	trace	-	-	-	-	-
3	β-pinene	968	974	1103	1118 ^6^	1.3	0.20	1.6	0.25	-	-	-	-
4	myrcene	986	988	1163	1166 ^7^	0.1	0.04	0.2	0.03	-	-	-	-
5	α-phellandrene	1001	1002	1159	1167 ^7^	trace	-	0.2	0.03	-	-	-	-
6	δ-3-carene	1003	1008	1142	1144 ^7^	trace	0.04	0.1	0.04	-	-	-	-
7	limonene	1023	1024	1194	1197 ^8^	0.5	0.34	0.7	0.27	-	-	-	-
8	*p*-mentha-2,4(8)-diene	1078	1085	1278	-	0.1	0.23	trace	-	-	-	-	-
9	*p*-cymene	1019	1020	1267	1281 ^6^	trace	-	trace	-	-	-	-	-
10	1,8-cineole	1025	1026	1201	1220 ^9^	0.3	0.20	0.5	0.26	0.6	0.20	0.4	0.06
11	linalool	1100	1095	1555	1556 ^6^	5.2	2.10	4.5	1.79	15.7	1.42	12.3	1.95
12	terpinen-4-ol	1172	1174	-	-	trace	-	trace	-	0.3	0.02	0.3	0.05
13	α-terpineol	1188	1186	-	-	0.2	0.09	trace	-	1,0	0.07	0.8	0.13
14	α-cubebene	1337	1348	1449	1461 ^7^	0.2	0.03	0.2	0.04	-	-	-	-
15	cyclosativene	1352	1369	1465	1522 ^10^	trace	-	trace	-	-	-	-	-
16	α-copaene	1363	1374	1479	1502 ^6^	3.7	0.74	3.9	0.73	-	-	-	-
17	β-bourbonene	1369	1387	1507	1517 ^7^	0.1	0.03	0.2	0.02	-	-	-	-
18	β-cubebene	1376	1387	1530	1542 ^11^	trace	-	trace	-	-	-	-	-
19	β-elemene	1379	1389	-	-	0.2	0.07	trace	-	-	-	-	-
20	α-gurjunene	1392	1400	1518	1530 ^12^	0.2	0.06	0.2	0.08	-	-	-	-
21	(*E*)-β-caryophyllene	1405	1417	1587	1589 ^13^	24.1	5.31	25.0	5.26	-	-	-	-
22	β-copaene	1414	1430	1581	1579 ^14^	0.3	0.06	0.3	0.02	-	-	-	-
23	β-ylangene	1421	1419	1562	1576 ^14^	0.1	0.03	0.1	0.04	-	-	-	-
24	aromadendrene	1425	1439	1632	1637 ^15^	trace	-	trace	-	-	-	-	-
25	β-gurjunene	1434	1431	1579	1590 ^7^	trace	-	0.4	0.09	-	-	-	-
26	6,9-guaiadiene	1440	1442	1658	1674 ^6^	0.1	0.03	trace	-	-	-	-	-
27	α-humulene	1454	1452	1668	-	11.6	1.80	12.0	1.70	-	-	-	-
28	*cis*-cadina-1(6),4-diene	1462	1465	1772	1788 ^14^	0.4	0.08	trace	-	-	-	-	-
29	*cis*-muurola-4(14),5-diene	1465	1461	-	-	0.4	0.20	trace	-	-	-	-	-
30	β-selinene	1472	1489	1706	1705 ^8^	3.3	0.47	3.1	0.38	-	-	-	-
31	α-selinene	1480	1498	1712	1725 ^14^	3.5	0.44	3.4	0.49	-	-	-	-
32	bicyclogermacrene	-	-	1722	1734 ^14^	trace	-	0.2	0.03	-	-	-	-
33	γ-muurolene	1483	1478	1697	1689 ^14^	0.6	0.30	0.5	0.24	-	-	-	-
34	α-amorphene	1486	1483	1679	1676 ^16^	0.7	0.23	0.4	0.03	-	-	-	-
35	valencene	1498	1496	1699	1728 ^14^	0.5	0.05	0.4	0.04	-	-	-	-
36	α-muurolene	1502	1511	1717	1734 ^6^	0.2	0.16	0.4	0.24	-	-	-	-
37	δ-cadinene	1506	1522	1750	1764 ^6^	2.8	0.19	2.8	0.33	-	-	-	-
38	*trans*-cadina-1,4-diene	1518	1533	-	-	0.1	0.08	-	-	-	-	-	-
39	*cis/trans*-calamenene	-	-	1824	1834 ^6^	trace	-	0.3	0.06	-	-	-	-
40	*trans*-cadina-1(6),4-diene	-	-	1882	-	trace	-	0.7	0.25	-	-	-	-
41	α-cadinene	1522	1537	1783	1769 ^6^	0.1	0.03	trace	-	-	-	-	-
42	α-calacorene	1525	1544	1907	1914 ^16^	0.2	0.03	0.2	0.07	-	-	-	-
43	β-calacorene	1546	1564	1948	1940 ^6^	0.3	0.17	trace	-	-	-	-	-
44	(*E*)-nerolidol	1558	1561	2045	2051 ^6^	4.0	1.10	3.7	0.97	-	-	-	-
45	caryophyllene oxide	1564	1582	1964	1989 ^6^	10.9	3.38	9.3	2.52	0.6	0.09	0.5	0.11
46	humulene epoxide II	1591	1608	2023	2047 ^14^	4.1	1.25	3.6	1.10	0.4	0.10	0.2	0.06
47	ledol	1583	1602	2016	2035 ^12^	0.2	0.16	0.2	0.10	-	-	-	-
48	spathulenol	-	-	2118	2126 ^6^	trace	-	1.3	0.52	0.4	0.07	0.3	0.05
49	caryophylla-4(12),8(13)-dien-5α-ol	1618	1639	2293	2301 ^17^	2.0	1.40	1.6	0.67	trace	-	0.2	0.05
50	α-muurolol (= torreyol)	1633	1644	2195	2142 ^12^	3.7	1.30	3.4	1.32	0.6	0.09	0.5	0.13
51	14-hyroxy-(*Z*)-caryophyllene	1657	1666	2378	-	2.0	0.89	1.9	0.65	-	-	-	-
52	amorpha-4,9-dien-2-ol	1671	1700	2358	-	0.6	0.40	0.4	0.16	-	-	-	-
	*Monoterpene hydrocarbons*					*4.4*		*5.8*		-		-	
	*Oxygenated monoterpenes*					*5.7*		*5.0*		*17.6*		*13.8*	
	*Sesquiterpene hydrocarbons*					*53.7*		*54.7*		-		-	
	*Oxygenated sesquiterpenes*					*27.5*		*25.4*		*2.0*		*1.7*	
	*Others*					-		-		-		-	
	*Total*					*91.3*		*90.9*		*19.6*		*12.1*	

^1^ Calculated linear retention indices (LRI); ^2^ reference linear retention indices according to [21]; ^3^ reference linear retention indices according to other literature; ^4^ percentage quantitative analysis; ^5^ standard deviation; trace < 0.1%; mw = molecular weight; ^6^ [22]; ^7^ [23]; ^8^ [24]; ^9^ [25]; ^10^ [26]; ^11^ [27]; ^12^ [28]; ^13^ [29]; ^14^ [30]; ^15^ [31]; ^16^ [32]; ^17^ [33].

**Table 2:** Enantioselective analysis of some chiral constituents of P. coruscans EO on 2,3-diethyl-6-*tert*-butyldimethylsilyl-β-cyclodextrin and 2,3-diacetyl-6-*tert*-butyldimethylsilyl-β-cyclodextrin.

Enantiomers	2,3-Diethyl-6-*tert*-Butyldimethylsilyl-β-Cyclodextrin			2,3-Diacetyl-6-*tert*-Butyldimethylsilyl-β-Cyclodextrin		
	LRI ^1^	Enantiomeric Distribution (%)	*e.e.* (%)	LRI ^1^	Enantiomeric Distribution (%)	*e.e.* (%)
(1*R*,5*R*)-(+)-α-pinene	927	15.2	69.6	975	20.0	60.0
(1*S*,5*S*)-(-)-α-pinene	928	84.8		970	80.0	
(1*R*,5*R*)-(+)-β-pinene	953	47.4	5.2	1041	46.4	7.2
(1*S*,5*S*)-(-)-β-pinene	961	52.6		1039	53.6	
(*R*)-(-)-α-phellandrene	1017	86.3	72.5	1092	89.1	78.2
(*S*)-(+)-α-phellandrene	1117	13.7		1158	10.9	
(*S*)-(-)-limonene	1052	35.7	28.6	1121	unseparable	-
(*R*)-(+)-limonene	1067	64.3				
(*R*)-(-)-linalool	1187	51.5	3.1	1384	50.9	1.8
(*S*)-(+)-linalool	1198	48.5		1386	49.1	

^1^ Linear retention index (LRI); *e.e.* = enantiomeric excess.
